# The Surgeon's Responsibilities

**Published:** 1904-06-11

**Authors:** 


### The Hospital.

A Journal of

The Medical Sciences and Hospital Administration.

Vol. XXXVI.—No. 924. JUNE 11, 1904.

### The Surgeon's Responsibilities.

The damages awarded to the plaintiff in the case of Byrne *v.* Thorne, which was concluded on Monday last, serve to illustrate the difficulty that the layman must of necessity experience when he is set to solve questions of a highly technical character. It will be remembered that the action was one for damages on account of alleged negligence in the performance of an abdominal operation, whereby a sponge was inadvertently left in the abdomen. It was acknowledged that the operation, which was a severe one, had been skilfully performed, but it was urged on behalf of the plaintiff that the operator had been careless in permitting a mistake to be made in the counting of the sponges. Both before and after the operation an experienced nurse, to whom the counting of the sponges was entrusted, on being twice questioned asserted both times that the number was correct. Relying on the nurse's statement, the surgeon stitched up the abdominal wound. A subsequent operation showed that a sponge had been left in the peritoneal cavity and that therefore the nurse's observations had been incorrect. Had the nurse been inexperienced and been paid by the surgeon the question of the latter's responsibility for the nurse's apparent carelessness would have been in a clearer light, but it appeared from the evidence that the nurse possessed a large experience, and was not in the pay of the surgeon. In view of the facts, the issue of the case really depended upon the operator's responsibility for the actions of his assistants; and in summing up the learned judge left the following question, among others, to the jury:—Was the defendant guilty of a want of due and reasonable care in regard to the counting, or superintending the counting, of the sponges? The jury, after deliberation, answered in the affirmative, and, finally, assessed the damages at £25.

The argument used with such effect on behalf of the plaintiff was that counting the sponges was a vital part of the operation, as it afforded the only means of knowing whether every one introduced into the abdomen had been removed, therefore the surgeon should either have counted the sponges himself or have seen that a second person counted them in addition to the nurse. It is quite possible that neither the learned judge himself nor the members of the jury have ever watched a severe abdominal operation; it is certain that without some technical instruction they would fail to appreciate the numberless details which absorb the operator's attention. Therefore it is, perhaps, not unnatural that they should be led into the fallacy of supposing that one individual is capable of personally attending to all the vital details of an extensive abdominal operation. We have watched many of the leading surgeons of the day performing laparotomy, and we cannot remember ever to have seen one himself count the sponges used, and were we to see a surgeon doing so it would strike us as being so unusual as to suggest that the individual had met with some previous misfortune which led him to adopt this uncommon plan. Indeed, if the surgeon must assure himself that every important detail which his assistants perform is correctly carried out he will require in future to possess, in addition to his other qualifications, at least half-a-dozen eyes and a corresponding number of independently acting brains in order to get through a single laparotomy within the space of 24 hours. The thing is impossible. The surgeon cannot personally supervise every essential detail. What would be thought of the sanity of anyone who should seriously put forward the view that every general in command of troops must, since he is responsible, personally verify every important piece of information imparted to him by his subordinate officers? The mental condition of a surgeon performing a severe abdominal operation is probably quite as complex as that of a commanding officer during an active engagement, and it is equally ridiculous in either case to hold that the individual in control ought not to act on observations and statements of his subordinates without first taking steps to verify the information received.

Nurses nowadays are highly trained in order that they may be able to bear such responsibility as falls to them, whether it be the taking of a temperature, the feeding of a typhoid patient, the preparation of a subject for operation, or any of the other innumerable offices upon which the patient's life will depend. If medical practitioners are to be held liable for mistakes made by nurses in performing these duties, the legal position of the doctor is even worse than we had previously supposed.

The fact that Miss Thorne gave her professional services to the plaintiff from charitable motives, and received no remuneration, will no doubt add to the sympathy that will be felt for her, though, as we had occasion to point out in a leading article last January, the liability of a medical man for any want of negligence which he may display in treating a patient is not removed by the fact that he has received no pecuniary reward for his services, and the plea, therefore, can only be advanced in mitigation of damages.

